# Full Genome Characterization of the *Culicoides-*Borne Marsupial Orbiviruses: Wallal Virus, Mudjinbarry Virus and Warrego Viruses

**DOI:** 10.1371/journal.pone.0108379

**Published:** 2014-10-09

**Authors:** Manjunatha N. Belaganahalli, Sushila Maan, Narender S. Maan, Ian Pritchard, Peter D. Kirkland, Joe Brownlie, Houssam Attoui, Peter P. C. Mertens

**Affiliations:** 1 Vector-borne Viral Diseases Programme, Institute for Animal Health, Pirbright, Woking, Surrey, United Kingdom; 2 Australian Animal Health Laboratory, CSIRO, Geelong, Victoria, Australia; 3 Elizabeth Macarthur Agricultural Institute, Camden, New South Wales, Australia; 4 Department of Pathology and Infectious Diseases, Royal Veterinary College, North Mymms, Hatfield, Herts, United Kingdom; Plymouth University, United Kingdom

## Abstract

Viruses belonging to the species *Wallal virus* and *Warrego virus* of the genus *Orbivirus* were identified as causative agents of blindness in marsupials in Australia during 1994/5. Recent comparisons of nucleotide (nt) and amino acid (aa) sequences have provided a basis for the grouping and classification of orbivirus isolates. However, full-genome sequence data are not available for representatives of all *Orbivirus* species. We report full-genome sequence data for three additional orbiviruses: Wallal virus (WALV); Mudjinabarry virus (MUDV) and Warrego virus (WARV). Comparisons of conserved polymerase (Pol), sub-core-shell ‘T2’ and core-surface ‘T13’ proteins show that these viruses group with other *Culicoides* borne orbiviruses, clustering with Eubenangee virus (EUBV), another orbivirus infecting marsupials. WARV shares <70% aa identity in all three conserved proteins (Pol, T2 and T13) with other orbiviruses, consistent with its classification within a distinct *Orbivirus* species. Although WALV and MUDV share <72.86%/67.93% aa/nt identity with other orbiviruses in Pol, T2 and T13, they share >99%/90% aa/nt identities with each other (consistent with membership of the same virus species *- Wallal virus*). However, WALV and MUDV share <68% aa identity in their larger outer capsid protein VP2(OC1), consistent with membership of different serotypes within the species - WALV-1 and WALV-2 respectively.

## Introduction

The orbiviruses are classified within the genus *Orbivirus*, family *Reoviridae*. They have icosahedral and non-enveloped virus-particles, containing a genome composed of ten linear pieces of dsRNA. The orbiviruses currently include 22 virus species (which also represent distinct ‘serogroups’) that have been recognised by the International Committee for the Taxonomy of Viruses (ICTV), as well as 14 unclassified isolates (some of which may represent additional species) [1]–[6]. The orbiviruses are primarily transmitted by ticks or haematophagous-insect-vectors (including mosquitoes, *Culicoides* [biting midges] and sand flies) and collectively have a wide host-range that includes domestic and wild ruminants, equines, marsupials, sloths, bats, birds and humans [2], [7], [8].

Orbiviruses, including Wallal virus (WALV), Warrego virus (WARV) and Eubenangee virus (EUBV) have previously been isolated from marsupials, and there is serological evidence for infections by Wongorr virus (WGRV) [7]. Evidence for a causal link between the orbiviruses and clinical disease in marsupials was provided by the isolation of WALV and WARV from animals affected by ‘kangaroo blindness’ in Australia during 1994/95 [9], [10]. Both viruses were isolated from Western Grey kangaroos (*Macropus fuliginosis*), red kangaroos (*Macropus rufa*), Eastern grey kangaroos (*Macropus giganteus*) and euros or wallaroo (*Macropus robustus*). Many thousands of kangaroos were affected in the states of New South Wales, South Australia and Victoria, suffering from non-suppurative choroiditis, retinal degeneration with inflammation and secondary degeneration of the optic nerve [9], [10]. A histopathology slide from a kangaroo with chorio-retinitis seen in the early 1970s, was found to have the same ‘structure’ as cases in 1994/95, suggesting periodic outbreaks of the disease [11]. Later in 1998–99, viruses belonging to the species *Eubenangee virus* were also isolated from wallabies affected by ‘Tammar Wallaby Sudden Death Syndrome’ (TSDS) [12], [13].

The species *Wallal virus* contains three distinct serotypes/strains: Wallal virus (WALV), Wallal K virus (WALKV) and Mudjinabarry virus (MUDV) [14]. WALV was first isolated in 1969 from adult *Culicoides* collected in Charleville, Queensland, Australia [15]. MUDV was isolated in September 1971 from *Culicoides marksi* collected in the Alligator Rivers area of the Northern Territory of Australia [16].

The species *Warrego virus* contains three distinct serotypes/strains: Warrego virus (WARV), Warrego K virus (WARKV) and Mitchell River virus (MRV). WARV virus was first isolated in 1970 from adult *Culicoides* collected in Charleville, Queensland, Australia [7], [15]. Mitchell River virus was isolated in April 1969 from *Culicoides spp*. trapped in the Mitchell River region in Australia [15].

The species *Eubenangee virus* contains four distinct viruses: Eubenangee virus (EUBV), Tilligerry virus (TILV), Pata virus (PATAV) and Ngoupe virus (NGOV) [2]. EUBV and TILV were isolated in Australia in 1963 and 1971 respectively from pools of mosquitoes [7]. They were also isolated from *Culicoides spp*. and antibodies to these viruses have been detected in cattle and marsupials [7], [17]. PATAV and NGOV were isolated in 1968 and 1974 respectively in the Central African Republic, also from pools of mosquitoes [7]. Nucleotide (nt) and amino-acid (aa) sequence comparisons indicate that PATAV represents a novel and distinct *Orbivirus* species [18].

The different species of *Culicoides* borne orbivirus (CBO) include *Bluetongue virus* (BTV) (the type species of the genus *Orbivirus*), *African horse sickness virus* (AHSV) and *Epizootic haemorrhagic disease virus* (EHDV), which are thought to include the most economically important orbiviruses. Complete genomes have been sequenced for representative isolates of BTV, EHDV, AHSV as well as some other CBO species including *Palyam virus* (PALV) and *Equine encephalosis virus* (EEV) (the members of which infect domesticated livestock) [19]–[23]. Although WALV, WARV and EUBV are also transmitted by adult *Culicoides*, [9], [12], [24] the full genome sequence of only one of these viruses – EUBV – has been reported [18].

Recent advances in RT-PCR and high throughput DNA sequencing methods have generated full-genome sequence data for multiple orbivirus isolates, helping to clarify the genetic relatedness and taxonomic status within and between different orbivirus species, including BTV, AHSV and EHDV [18], [19], [25]–[28]. Comparisons of nucleotide (nt) and amino acid (aa) sequence data now provide a basis for molecular epidemiology studies and for grouping distinct orbivirus isolates into serotypes and topotypes [19], [26], [29], [30].

As part of a programme to generate representative data for all *Orbivirus* species, we report full genome nucleotide sequences for WALV, MUDV and WARV, and present a comparison to isolates from other *Orbivirus* species as an aid to help identify novel orbivirus isolates. The full-genome sequences of WALV and MUDV indicate that they represent different serotypes within the *Wallal virus* species and that WARV represents a distinct species within the genus *Orbivirus*.

## Materials and Methods

### Viruses

The viruses used in this study [AUS1978/09 (WALV), AUS1982/03 (MUDV) and AUS1969/01 (WARV)] were obtained from the Orbivirus Reference Collection (ORC) at The Pirbright Institute. These viruses were originally taken from naturally infected animals by qualified veterinarians, as part of normal diagnostic testing procedures in the respective countries. MUDV was propagated in BHK-21 cells (clone 13 obtained from European Collection of Animal cell Cultures (ECACC – 84100501), while WALV and WARV were grown in BSR cells (a clone of BHK) [35] or BHK cells, in Dulbecco's minimum essential medium (DMEM) supplemented with antibiotics (100 units/ml penicillin and 100 µg/ml streptomycin) and 2 mM glutamine. Infected cell cultures were incubated until they showed widespread (100%) cytopathic effects (CPE). Viruses were harvested, aliquoted and used for the dsRNA extraction, or stored in the orbivirus reference collection (ORC) at −80°C.

### Preparation of viral dsRNA

Intact genomic dsRNA was extracted from WALV, MUDV and WARV infected cell cultures using a guanidinium isothiocyanate extraction procedure as described by Attoui et al [36]. Briefly, the infected cell pellet was lysed in 1 ml of commercially available TRIZOL reagent (Invitrogen), 0.2 volume of chloroform was added, mixed by vortexing and the mixture incubated on ice for 10 min. The supernatant containing total RNA was separated from cellular debris and DNA by centrifuging at 10,000 x*g* for 10 min at 4°C. Single stranded RNA (ssRNA) was removed by 2 M LiCl precipitation at 4°C overnight, followed by centrifugation at 10,000 x*g* for 5 min. An equal volume of isopropanol containing 750 mM ammonium acetate was added to the supernatant, then mixed and the viral dsRNA was allowed to precipitate for a minimum of 2 hours at −20°C. The dsRNA was pelleted by centrifugation at 10,000 x*g* for 10 min, washed with 70% ethanol, air dried and suspended in nuclease free water (NFW). The RNA was either used immediately or stored at −20°C.

### Reverse transcription of dsRNA and PCR amplification of cDNAs

The genome segments of WALV, MUDV and WARV were reverse-transcribed using a ‘full-length amplification of cDNA' (FLAC) technique described by Maan et al [37]. Briefly, a 35 base self-priming oligonucleotide ‘anchor-primer’ with a phosphorylated 5′ terminus was ligated to the 3′ ends of the viral dsRNAs using the T4 RNA ligase, followed by reverse transcription using RT system (Promega). The resulting cDNAs were amplified using complementary primers to the anchor primer. The resulting cDNA amplicons were analyzed by agarose gel electrophoresis. For cloning purposes, a high fidelity KOD polymerase enzyme (Novagen) was used in the PCR.

### Cloning and sequencing of cDNAs

Amplified cDNAs were purified and cloned. MUDV genes were cloned into the Strataclone blunt-end PCR cloning vector ‘pSC-B-amp/kan’ supplied in the StrataClone Blunt PCR cloning kit (Stratagene). WALV (only Seg-2) and WARV amplicons were cloned into the ‘pCR-Blunt’ vector supplied with the Zero Blunt PCR Cloning Kit (Invitrogen). Recombinant plasmid-vectors containing MUDV inserts were transformed into Solopack competent cells (Agilent Technologies), while WALV and WARV plasmids were transformed into One Shot TOP10 competent cells supplied with the respective cloning kits. Clones containing relevant inserts were identified by touch PCR using M13 universal primers. Plasmids were extracted from the clones identified using the QIAprep Spin MiniPrep Kit (Qiagen). The plasmids and PCR products were sequenced using an automated ABI 3730 DNA sequencer (Applied Biosystems). Some of the MUDV sequencing primers detected other segments of WALV and using the sequences generated, running primers were designed to obtain full sequence for the remaining nine segments of WALV.

### Sequence analysis and phylogenetic tree construction

‘Raw’ ABI sequence data were assembled into ‘contigs’ using the SeqManII sequence analysis package (DNAstar version 5.0). The ORFs of WALV, MUDV and WARV were identified and translated into aa sequences for further analysis using EditSeq (DNAstar version 5.0). The putative function of each protein was identified by BlastX comparisons to homologous orbivirus (e.g. BTV) proteins in GenBank (http://blast.ncbi.nlm.nih.gov/Blast.cgi?CMD=Web&PAGE_TYPE=BlastHome). Multiple alignments of consensus sequences were performed using ClustalX (Version 2.0) [38], Clustal Omega (http://www.ebi.ac.uk/Tools/msa/clustalo/) and MAFFT [39] to ensure proper alignment. Aligned protein sequences were back translated to nucleotide sequences using DAMBE [40]) or RevTrans 1.4 server available online (http://www.cbs.dtu.dk/services/RevTrans/) for further nucleotide analysis. The best fit amino acid (aa) and nucleotide (nt) models for Maximum likelihood (ML) analysis were determined using ProtTest 3.0 and jModeltest respectively [41], [42]. The models were also determined using MEGA 5 software. The consensus or simplest model given by Akaike Information Criterion (AIC) and Bayesian Information Criterion (BIC) was selected for ML tree construction. The aa model rtREV (G+F) was selected for T2 tree and rtREV (I+G+F) model was selected for both VP1 and T13 proteins. One nt model, GTR (+G) was used to construct ML trees for all three genes. All phylogenetic trees construction and pairwise distance calculations using p-distance parameter were performed using MEGA 5 [43], [44]. GenBank nucleotide accession numbers for the sequences used for analysis and phylogenetic studies are listed in the Table S1 in File S1.

## Results

### Virus propagation and genomic-segment migration-pattern (electropherotype)

**Figure 1:**
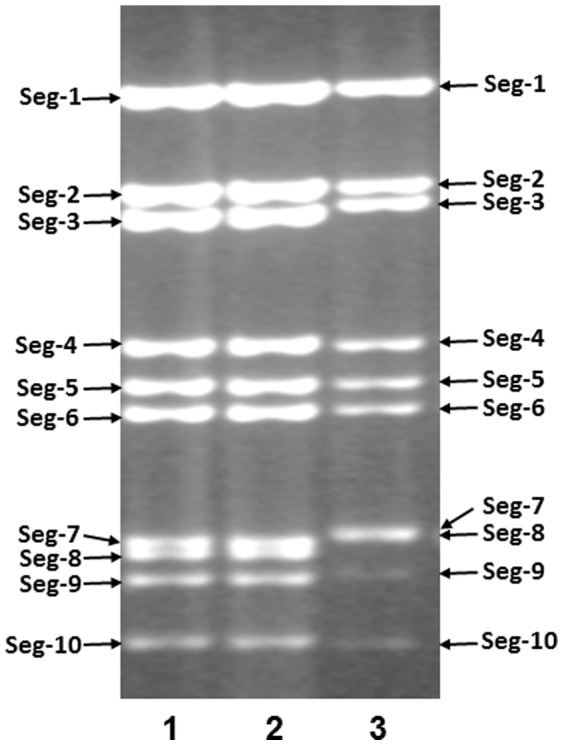
Agarose gel (1%) electrophoretic profile of dsRNAs of Wallal virus (WALV), Mudjinabarry virus (MUDV) and Warrego viruses (WARV). 1 =  WALV (AUS1978/09); 2 =  MUDV (AUS1982/03); 3 = WARV (AUS1969/01).

WALV (isolate - AUS1978/09), MUDV (isolate AUS1982/03) and WARV (isolate AUS1969/01) all induced severe cytopathic effects (CPE) in BHK and BSR (a clone of BHK cells) cell monolayers at 48–72 hours post infection. When analysed by 1% agarose gel electrophoresis (AGE), the genomic dsRNA of these viruses migrated as distinct groups, consisting of 3 large segments, 3 medium size segments, 3 small segments, and 1 very small segment (giving a 3-3-3-1 pattern) (Figure 1). This pattern is similar to that observed in other CBOs (including BTV, EHDV and EUBV) [18], [37]. WALV and MUDV generated identical genome segment migration patterns (‘electropherotype’) during AGE, as expected for viruses belonging to the same virus species (Figure 1) [2], [37]. In contrast, WARV showed clear differences in the migration of Seg-3, -7, -8 and -9 (Lane 3 in Figure 1) confirming that it belongs to a different virus species.

### Genome sequence analyses of WALV (AUS1978/09), MUDV (AUS1982/03) and WARV (AUS1969/01)

Full length sequences of Seg-1 to Seg-10 of WALV (AUS1978/09), MUDV (AUS1982/03) and WARV (AUS1969/01) have been submitted to GenBank with accession numbers KJ495745 to KJ495754; KJ495765 to KJ495774 and KJ495755 to KJ495764 respectively. These data were compared to other orbiviruses (accession numbers for the data used are given in Table S1 in File S1). The total genome size of WALV, MUDV and WARV is 19,387 bp, 19,382 bp and 19,428 bp respectively, with segments ranging from 3959 bp to 862 bp (3964 bp to 838 bp in WARV), encoding proteins of 1306 aa to 87 aa (1303 aa to 78 aa in WARV) (Table 1 and 2). As suggested by the electropherotype analysis (Figure 1), most of the individual genome segments of WALV and MUDV, with the exception of Seg-2 (VP2) and Seg-8 (NS2(ViP)) have identical sizes with similar ORFs, identical start and stop positions generating proteins of the same length (Table 1).

**Table 1:** Characteristics of dsRNA genome segments and proteins of the WALV (AUS1978/09) and MUDV (AUS1982/03) viruses.

Virus/Segment	Segment length (bp)	Protein encoded	Protein length (aa)	Protein mass (kDa)	ORFs bp (including stop codon)	5′ NCRs	3′ NCR	5′ conserved Termini	3′ conserved Termini	Accession numbers
**WALV**										
Seg-1	3959	Pol	1306	151.14	13–3933	12	29	5′-GUAUA	ACACUUAC-3′	KJ495745
Seg-2	3003	OC1	983	114.74	18–2969	17	37	5′-GUAUA	AAGCUUAC-3′	KJ495746
Seg-3	2783	T2	899	103.68	20–2719	19	67	5′-GUAUA	ACACUUAC-3′	KJ495747
Seg-4	1985	CaP	644	75.61	11–1945	10	43	5′-GUAUA	ACACUUAC-3′	KJ495748
Seg-5	1790	TuP	557	64.82	31–1704	30	89	5′-GUAUA	ACACUUAC-3′	KJ495749
Seg-6	1660	OC2	530	59.91	32–1624	31	39	5′-GUAUA	ACACUUAC-3′	KJ495750
Seg-7	1162	T13	350	38.06	19–1071	18	94	5′-GUAUA	ACACUUAC-3′	KJ495751
Seg-8	1132	ViP	341	38.66	68–1093	67	42	5′-GUAUA	ACACAUAC-3′	KJ495752
Seg-9	1051	Hel	329	36.17	17–1006	16	48	5′-GUAUA	ACACAUAC-3′	KJ495753
		NS4	87	10.49	123–386					
Seg-10	862	NS3	246	26.94	19–759	18	106	5′-GUAAA	AAACCUAC-3′	KJ495754
		NS3a	211	22.78	124–759					
**Total**	**19387**						**Consensus**	**5′-GUA^U^/_A_A**	**A^C^/_A_^A^/_G_C^U^/_A/C_UAC-3′**	
**MUDV**										
Seg-1	3959	Pol	1306	151.16	13–3933	12	29	5′-GUAUA	ACACUUAC-3′	KJ495765
Seg-2	2997	OC1	981	114.87	18–2963	17	37	5′-GUAUA	AAGCUUAC-3′	KJ495766
Seg-3	2783	T2	899	103.69	20–2719	19	67	5′-GUAUA	ACACUUAC-3′	KJ495767
Seg-4	1985	CaP	644	75.23	11–1945	10	43	5′-GUAUA	ACACAUAC-3′	KJ495768
Seg-5	1789	TuP	557	64.77	31–1704	30	88	5′–GUAUA	ACACUUAC-3′	KJ495769
Seg-6	1660	OC2	530	59.76	32–1624	31	39	5′-GUAUA	ACACUUAC-3′	KJ495770
Seg-7	1163	T13	350	38.06	19–1071	18	95	5′-GUAUA	ACACUUAC-3′	KJ495771
Seg-8	1133	ViP	341	38.72	69–1094	68	42	5′-GUAUA	ACACAUAC-3′	KJ495772
Seg-9	1051	Hel	329	36.11	17–1006	16	48	5′-GUAUA	ACACAUAC-3′	KJ495773
		NS4	87	10.43	123–386					
Seg-10	862	NS3	246	26.95	19–759	18	106	5′-GUAUA	AAACCUAC-3′	KJ495774
		NS3a	211	22.79	124–759					
**Total**	**19382**						**Consensus**	**5′-GUAUA**	**A^C^/_A_^A^/_G_C^U^/_A/C_UAC-3′′**	

**Table 2:** Characteristics of dsRNA genome segments and proteins of WARV (AUS1969/01) virus.

Segment	Segment length (bp)	Protein encoded	Protein length (aa)	Protein mass (kDa)	ORFs bp (including stop codon)	5′ NCRs	3′ NCR	5′ conserved Termini	3′ conserved Termini	accession numbers
**1**	3964	Pol	1308	150.69	12–3938	11	29	5′-GTAAAA	ACTTAC-3′	KJ495755
**2**	2992	OC1	979	115.48	18–2957	17	38	5′-GTAAAA	ACTTAC-3′	KJ495756
**3**	2845	T2	928	106.79	15–2801	14	47	5′-GTAAAA	ACATAC-3′	KJ495757
**4**	1965	CaP	639	74.65	9–1928	8	40	5′-GTAAAA	ACATAC-3′	KJ495758
**5**	1771	TuP	530	62.17	96–1688	95	86	5′-GTAAAA	AACTAC-3′	KJ495759
**6**	1652	OC2	528	59.45	29–1615	28	40	5′-GTAAAA	ACATAC-3′	KJ495760
**7**	1185	ViP	370	42.22	30–1142	29	46	5′-GTAAAA	ACTTAC-3′	KJ495762
**8**	1165	T13	350	38.44	19–1071	18	97	5′-GTAAAA	ACATAC-3′	KJ495761
**9**	1051	Hel	330	36.82	16–1008	15	46	5′-GTAAAA	ACATAC-3′	KJ495763
		NS4	78	9.72	113–349					
**10**	838	NS3	250	27.79	21–773	20	68	5′-GTAAAATT	ACTTAC-3′	KJ495764
		NS3a	219	24.19	114–773					
**Total**	**19428**						**Consensus**	**5′-GTAAAA**	**A^C^/_A_^A^/_T/C_TAC-3’**	

The different genome segments of WALV and MUDV have the same conserved termini, sharing five fully conserved nucleotides at their 5′ and 3′ends (+ve: 5′-GUA^U^/_A_A………..A^C^/_A_^A^/_G_C^U^/_A/C_UAC-3′). WARV shares six fully conserved nucleotides at the 5′ ends and four at the 3′ ends (+ve: 5′-GUAAAA………..A^C^/_A_^A^/_U/C_UAC-3′). Two terminal nucleotides at the 5′ end and three nucleotides at the 3′ end (5′-GU……UAC-3′) of all three viruses are characteristic of the genus *Orbivirus*, and the first and last two nucleotides represent inverted complements (http://www.reoviridae.org/dsRNA_virus_proteins/CPV-RNA-Termin.htm).

The G+C content of the WALV, MUDV and WARV genomes are 39.14%, 39.41% and 38.99% respectively, well within the range previously identified for insect-borne orbiviruses (IBOs) (36.66% in PHSV to 45.86% in EEV) but less than tick-borne orbiviruses (TBOs) (51.93% in St Croix River virus (SCRV) to 57.29% in Great Island virus (GIV)) [5].

Like other orbiviruses, most genome segments of WALV, MUDV and WARV have shorter 5' than 3' terminal non-coding regions (NCRs). However, Seg-8 (encoding the viral inclusion body protein NS2) of WALV and MUDV, and Seg-5 (encoding the viral tubule protein NS1) of WARV have longer 5′ NCRs (Table 1). Exceptions were also observed in Umatilla virus (UMAV), Corriparta virus (CORV) and GIV, although the significance of these variations is uncertain [5]. Collectively the upstream and downstream terminal NCRs represent 4.29%, 4.3% and 4.08% of the WALV, MUDV and WARV genomes respectively. Comparisons with other CBOs showed similar or higher percentages, while the mosquito-borne orbiviruses (MBOs) and TBOs show higher values [5].

The coding assignments of the genome segments of WALV and MUDV (Table 1) are similar to those of BTV [45], [46]. The coding assignments of WARV are also similar, with the exception of Seg-7 and Seg-8 (coding for ViP and VP7 respectively), which have reversed their relative order of migration/size (Table 2). Most of the genome segments are monocistronic, encoding proteins from single open reading frames (ORF). However, Seg-10 of all three viruses has two in-frame initiation sites, at or near the start of the same ORF leading to translation of NS3 and NS3a (Table 1). Recently, a novel non-structural protein ‘NS4’ (which varies between 10 kDa to 22.5 kDa, depending on *Orbivirus* species), encoded by a downstream ORF (+2 frame) on Seg-9 (which also encodes VP6-Helicase) was identified in BTV and several other orbiviruses [33], [47], [48]. Similarly WALV, MUDV and WARV have a downstream ORF (+2 frame) on Seg-9 that encodes NS4.

Most of the WALV and MUDV ORF's have a ‘strong’ Kozak sequence (RNN**AUGG**), except for the first ORFs of Seg-9 and Seg-10, which have ‘weak’ (UAG**AUG**U) or ‘moderate’ (GUC**AUG**U) contexts respectively, potentially allowing read-through of the upstream initiation codon, enhancing expression of NS4 and NS3a respectively. Similarly, most ORF's of the WARV genome-segments have a strong (RNN**AUG**G) to moderate Kozak sequence [Seg-4 (AUC**AUG**C) and Seg-5 (UCU**AUG**A)], except Seg-9 (UGG**AUG**U) and Seg-10 (GUC**AUG**C), both of which have a weak ‘context’.

Weak or moderate Kozak sequences have previously been observed in several of the genome segments of other orbiviruses, although they still appear to be translated effectively [18], [33]. The down-stream NS4 ORF of Seg-9 in WALV and MUDV has a strong Kozak context (**A**AG**AUGG**) encoding an 87 aa protein, and in WARV has a moderate Kozak sequence (GGG**AUG**C) encoding 78 aa protein. In all three viruses WALV, MUDV and WARV, the second initiation site (for NS3a) in Seg-10 has the strong Kozak context (A^A^/_C_G**AUG**G).

### Phylogenetic relationships of Seg-1/VP1(Pol)

**Figure 2:**
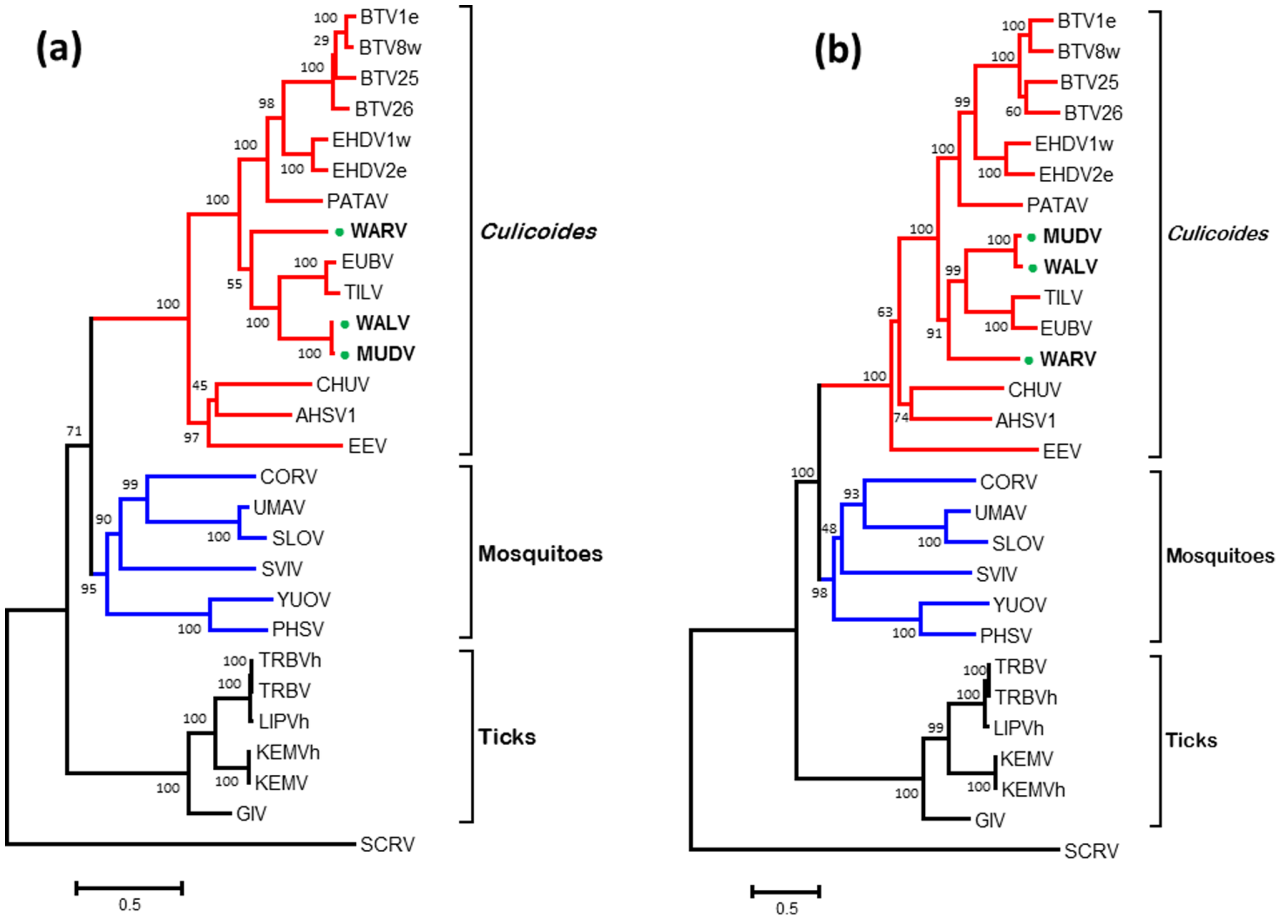
Maximum likelihood (ML) trees showing phylogenetic comparisons of T2 protein amino acid sequences (a) and nucleotide sequences of Seg-3 (b) of WALV, MUDV and WARV, aligned with those of other *Orbivirus* species. One thousand bootstrap replicates were used for construction of the phylogenetic trees. The WALV, MUDV and WARV isolates characterised in this study are marked with green circle. BTV =  Bluetongue virus; AHSV =  African Horse sickness virus; EHDV =  Epizootic Haemorrhagic Disease Virus; CHUV =  Chuzan virus; EUBV =  Eubenangee virus; TILV =  Tilligerry virus; PATAV =  Pata virus; EEV =  Equine encephalosis virus; WARV =  Warrego virus; WALV =  Wallal virus; MUDV =  Mudjinbarry virus; CORV =  Corriparta virus; UMAV =  Umatilla virus; SLOV =  Stretch Lagoon orbivirus; SVIV =  Sathuvachari virus; YUOV =  Yunnan orbivirus; PHSV =  Peruvian horse sickness virus; TRBV =  Tribec virus; TRBVh =  another isolate of Tribec virus; LIPV =  Lipovnik virus; LIPV =  Lipovnik virus isolate CzArLip 91; KEMV =  Kemerovo virus; KEMVh =  Kemerovo virus isolate EgAn 1169-61; GIV =  Great Island virus; SCRV =  St Croix river virus. Virus isolates and accession numbers of polymerase sequences used for comparative analysis are listed in Table S1 in File S1. ‘e’ and ‘w’ after serotype number indicate eastern and western strains, respectively.

The conserved nature of the orbivirus RNA dependent RNA polymerase (Pol) (encoded by Seg-1) has previously been used to help classify orbivirus isolates at the genus and species level based on sequence comparisons and identity levels [18], [25], [27], [31]. Phylogenetic comparisons of Seg-1/VP1(Pol) from WALV, MUDV and WARV with other orbiviruses (Figure 2), showed three groups containing the CBOs, MBOs and TBOs. In each case WALV, MUDV and WARV grouped with the CBOs.

Seg-1/VP1(Pol) of WALV and MUDV share 99.16/93.57 aa/nt identity, confirming that they belong to the same *Orbivirus* species. In comparisons to other orbiviruses they show a maximum of 70.96%/66.97% aa/nt identity with EUBV and a minimum of 36.08%/43.55% with SCRV (Table 3). VP1(Pol) of WARV shared up to a maximum of 65.41%/63.72% aa/nt identities with EHDV-2w, indicating its distinctiveness from other *Orbivirus* species within the CBOs.

**Table 3:** Percent amino acid and nucleotide identities of WALV (AUS1978/09), MUDV (AUS1982/03) and WARV (AUS1969/01) with other orbiviruses in VP1(Pol), T2 and T13 protein/genes.

Orbivirus	WALV						MUDV						WARV					
	VP1		T2		T13		VP1		T2		T13		VP1		T2		T13	
	aa	nt	aa	nt	aa	nt	aa	nt	aa	nt	aa	nt	aa	nt	aa	nt	aa	nt
**Vector: CBOs**																		
**BTV1e**	63.28	61.83	68.63	64.81	48.71	54.35	63.36	61.83	68.74	65.15	48.71	54.06	62.95	61.87	64.15	63.52	53.30	57.21
**BTV8w**	63.59	62.38	68.97	66.37	49.00	52.34	63.74	62.07	69.08	66.07	49.00	52.91	63.03	62.29	64.59	62.89	53.30	56.45
**BTV25**	62.74	62.48	67.52	64.59	49.57	53.39	62.89	62.71	67.63	65.18	49.57	53.10	62.34	62.98	63.60	64.71	53.58	57.50
**BTV26**	63.28	61.38	67.41	65.55	50.14	53.20	63.20	61.59	67.52	65.04	50.14	52.72	63.18	62.16	64.59	62.45	54.15	57.59
**EHDV1w**	62.82	63.54	69.71	65.63	49.57	54.25	62.89	63.31	69.82	65.81	49.57	54.35	65.10	63.95	64.07	63.52	**56.45**	**59.41**
**EHDV2e**	62.51	63.28	69.82	66.07	51.00	54.15	62.59	62.84	69.93	66.78	51.00	54.15	**65.41**	63.72	64.40	62.85	56.16	58.26
**PATAV**	64.54	63.28	68.71	65.70	55.17	58.81	64.46	63.49	68.82	65.48	55.17	59.00	64.49	63.52	65.29	64.11	50.57	57.18
**EUBV**	70.96	66.77	72.86	66.89	57.71	60.67	70.96	66.97	72.86	67.33	57.71	59.90	64.42	63.30	**67.33**	64.41	52.00	58.57
**TILV**	69.73	66.21	72.41	67.89	58.57	60.00	70.04	66.39	72.41	67.93	58.57	59.43	64.04	63.96	67.11	64.44	52.57	58.86
**WALV**	---	---	---	---	---	---	**99.16**	**93.57**	**99.78**	**90.36**	**100.00**	**97.71**	64.37	65.29	66.30	**65.55**	48.29	55.24
**MUDV**	**99.16**	**93.57**	**99.78**	**90.36**	**100.00**	**97.71**	---	---	---	---	---	---	64.60	**65.36**	66.41	65.04	48.29	55.33
**WARV**	64.37	65.29	66.30	65.55	48.29	55.24	64.60	65.36	66.41	65.04	48.29	55.33	---	---	---	---	---	---
**AHSV1**	58.80	61.59	61.40	61.92	46.70	53.39	58.57	60.80	61.29	62.48	46.70	53.30	58.56	60.27	59.62	60.62	44.13	51.96
**CHUV**	57.63	60.21	60.29	61.36	39.94	49.33	57.78	60.44	60.40	62.25	39.94	49.43	56.03	59.53	57.25	60.43	41.67	52.59
**EEV**	52.65	56.16	50.83	56.17	40.29	48.86	52.65	56.32	50.83	55.28	40.29	49.24	51.53	55.04	50.28	54.38	41.71	49.52
**Vector: MBOs**																		
**PHSV**	48.18	55.10	36.30	48.85	21.78	42.02	48.02	55.20	36.41	48.96	21.78	41.64	47.87	55.12	35.94	49.55	21.20	40.11
**YUOV**	46.49	54.11	38.20	49.11	23.78	40.78	46.41	53.93	37.97	50.37	23.78	40.40	45.41	53.59	35.92	47.90	20.06	38.11
**UMAV**	47.86	54.04	36.01	47.97	24.07	38.11	47.54	53.78	36.12	48.20	24.07	38.49	48.56	54.53	35.48	47.97	22.64	38.11
**SLOV**	47.08	53.78	36.68	47.79	---	---	46.92	53.73	36.79	48.46	---	---	48.09	53.05	35.48	45.34	---	---
**CORV**	48.16	53.87	37.86	48.52	23.78	38.78	48.16	54.21	37.75	48.29	23.78	38.68	47.31	53.19	35.64	47.84	25.21	40.59
**SVIV**	46.87	52.57	37.75	49.33	25.00	41.00	47.10	52.85	37.75	49.89	25.00	40.80	47.22	54.04	35.48	47.82	22.70	36.69
**Vector: TBOs**																		
**TRBV**	45.08	49.33	35.86	44.02	25.21	38.68	44.93	48.81	35.97	43.76	25.21	38.20	45.29	49.03	35.14	43.13	24.07	37.15
**KEMV**	45.91	49.74	35.08	44.39	24.93	37.25	45.83	49.74	35.19	43.65	24.93	37.54	45.80	49.44	35.91	43.35	23.50	37.34
**GIV**	45.28	48.90	36.53	45.58	24.93	37.25	45.13	49.11	36.64	44.69	24.93	37.15	46.98	49.41	36.46	43.46	23.21	35.24
**Vector: Tick associated**																		
**SCRV**	36.08	43.55	23.49	37.78	19.53	34.79	36.08	43.73	23.49	37.86	19.53	34.69	37.30	44.60	24.40	37.17	20.41	32.07

BTV =  Bluetongue virus; BTV1e  =  BTV-1 eastern topotype; BTV8w  =  BTV-8 western topotype; EHDV =  Epizootic haemorrhagic disease; EHDV1w  =  EHDV-1 western topotype; EHDV2e  =  EHDV-2 eastern topotype; PATAV  =  Pata virus; EUBV  =  Eubenangee virus; TILV  =  Tilligerry virus; WALV  =  Wallal virus; MUDV  =  Mudjinbarry virus; WARV  =  Warrego virus; AHSV  =  African horse sickness virus; CHUV  =  Chuzan virus; EEV  =  Equine encephalosis virus; PHSV  =  Peruvian horse sickness virus; YUOV  =  Yunnan orbivirus; UMAV  =  Umatilla virus; SLOV  =  Stretch Lagoon orbivirus; CORV  =  Corriparta virus; SVIV  =  Sathuvachari virus; TRBV  =  Tribec virus; KEMV  =  Kemorovo virus; GIV  =  Great Island virus; SCRV  =  St Croix river virus.The highest identities were written in bold font.

### Phylogenetic relationships of the subcore-shell ‘T2’ protein

**Figure 3:**
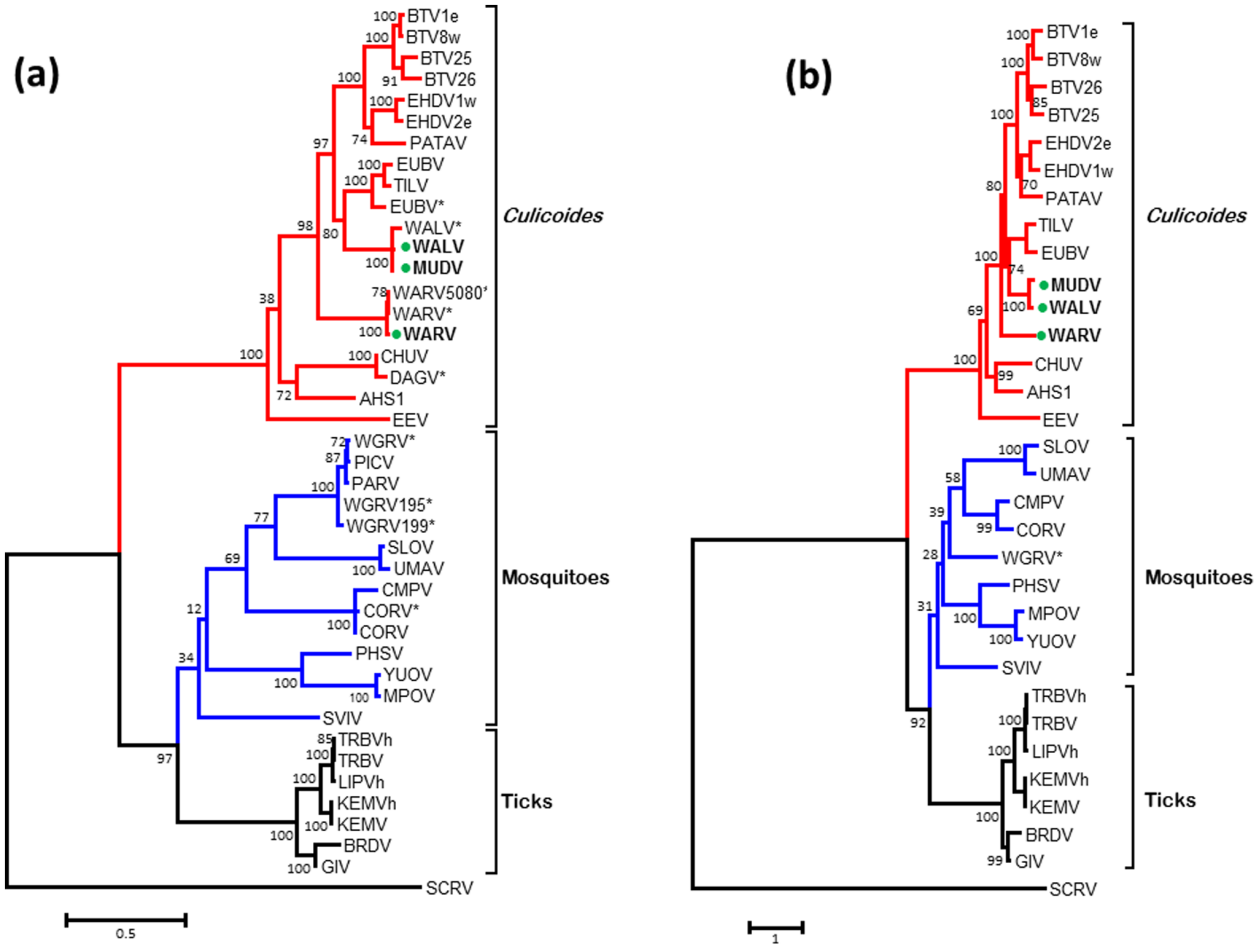
Maximum likelihood (ML) trees showing phylogenetic comparisons of the VP1 protein (a) and nucleotide sequences of Seg-1 (b) of WALV, MUDV and WARV, aligned with those of other *Orbivirus* species. One thousand bootstrap replicates were used for construction of the phylogenetic trees. The WALV, MUDV and WARV isolates characterised in this study are marked with green circle. WGRV =  Wongorr virus; DAGV =  D'Aguilar virus; PICV =  Picola virus; PARV =  Paroo river virus; CMPV =  California mosquito pool virus; MPOV =  Middle point orbivirus; BRDV =  Broadhaven virus. Further abbreviations used in this figure are same as mentioned in Figure 2. Virus isolates and accession numbers of polymerase sequences used for comparative analysis are listed in Table S1 in File S1. ‘e’ and ‘w’ after serotype number indicate eastern and western strains, respectively.

BlastX analyses confirmed that the subcore shell T2 protein - VP3 - is encoded by Seg-3 of WALV, MUDV and WARV. Phylogenetic trees constructed for the T2 protein of different orbiviruses (Figure 3 and Figure S1 in File S1) showed that the CBOs (including BTV, AHSV, EHDV, WALV, EUBV, WARV and PALV) form a single group, in which T2(VP3) is encoded by Seg-3. The TBOs (GIV) and the MBOs (CORV, WGRV, Peruvian horse sickness virus [PHSV] and Yunnan orbivirus [YUOV]), form two further groups, in both of which the subcore T2(VP2) is encoded by Seg-2. The T2 protein (encoded by Seg-2) of SCRV branches separately, representing a more distantly related orbivirus from ticks.

The T2 protein/gene of WALV and MUDV clustered together, sharing 99.78%/90.36% aa/nt identities (Table 3), confirming that they belong to the same *Orbivirus* species (Figure 3). Comparisons to other orbiviruses showed a maximum of 72.86%/67.89% aa/nt identities with EUBV, down to 23.49%/37.44% with SCRV supporting classification of WALV and MUDV within a separate virus species. In contrast, WARV branched separately from other orbiviruses, sharing maximum aa/nt identities of 67.33%/65.55% with EUBV/WALV and a minimum of 24.4%/37.17% with SCRV (Table 3) confirming its classification within a distinct species.

### Phylogenetic comparisons of Seg-7/VP7(T13)

**Figure 4:**
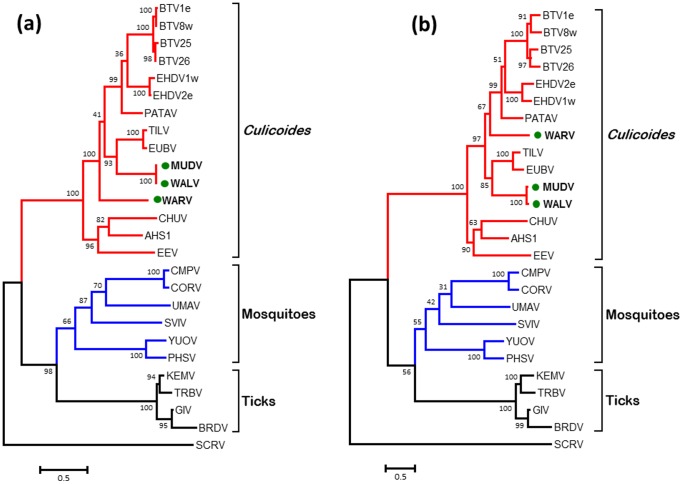
Maximum likelihood (ML) trees showing phylogenetic comparisons of the T13 protein (a) and gene (b) sequences of WALV, MUDV and WARV, aligned with those of other *Orbivirus* species. One thousand bootstrap replicates were used for construction of the phylogenetic trees. The WALV, MUDV and WARV isolates characterised in this study are marked with green circle. Abbreviations used in this figure are same as mentioned in Figure 2 and 3. Virus isolates and accession numbers of polymerase sequences used for comparative analysis are listed in Table S1 in File S1. ‘e’ and ‘w’ after serotype number indicate eastern and western strains, respectively.

The core surface protein VP7(T13) is the most abundant of the orbivirus structural proteins and is a strongly immuno-dominant serogroup/species-specific antigen [49]. The ML trees constructed for VP7(T13) protein and gene sequences of WALV, MUDV, WARV and other orbiviruses (Figure 4) exhibited similar topology to the T2 and VP1(Pol) trees (Figures 2 and 3) with three distinct ‘vector-groups’ (CBOs, MBOs and TBOs). WALV and MUDV again grouped together, while WARV branched separately within the CBOs. When compared to other orbiviruses, the VP7(T13) proteins/genes of WALV and MUDV (which share 100%/97.71% aa/nt identity) showed a maximum of 58.57%/60.67% aa/nt identity with EUBV and TILV. In contrast WARV again showed maximum aa/nt identity with EHDV (56.45%/59.45%). WALV and WARV share 39.94% to 58.57% aa sequence identity with the other CBOs; 20.06% to 25.21% aa identity with MBOs; 21.04% to 25.21% aa identity with the TBOs; but only 19.53% to 20.41% aa identity with the more distantly related SCRV (Table 3).

### Phylogenetic comparisons of Seg-2/VP2(OC1)

**Figure 5:**
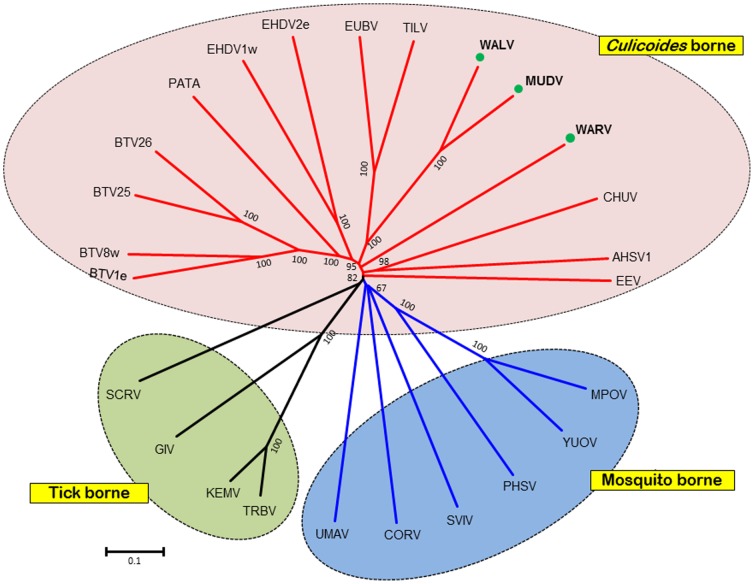
Unrooted neighbour-joining tree showing the relationships between amino acid sequences of OC1 protein of different orbiviruses. One thousand bootstrap replicates were used for construction of the phylogenetic trees. The VP2(OC1) protein of WALV, MUDV and WARV isolates characterised in this study are marked with green circle. CBOs =  *Culicoides* borne orbiviruses; MBOs =  Mosquito borne orbiviruses; TBOs =  Tick borne orbiviruses. Abbreviations used for viruses in this figure are same as mentioned in Figure 2 and 3. Virus isolates and accession numbers of polymerase sequences used for comparative analysis are listed in Table S1 in File S1. ‘e’ and ‘w’ after serotype number indicate eastern and western strains, respectively.

Orbivirus outer capsid protein (OC1) determines virus serotype and is highly variable, both in sequence identity levels and in size. VP2(OC1) of the CBOs is encoded by Seg-2 (VP2), by Seg-3 (VP3) of the MBOs and by Seg-4 (VP4) of the TBOs [5], [18], [33]. In WALV, MUDV and WARV viruses, VP2(OC1) is encoded by Seg-2, consistent with *Culicoides* transmission. An unrooted NJ phylogenetic tree constructed for OC1 of different orbiviruses (Figure 5) identified three major clusters, again corresponding to their ‘vector groups’ in a manner similar to the trees for VP1, T2 and T13 proteins (Figures 2, 3 and 4). These groupings suggest that despite a high level of aa sequence variation in response to immunological pressure from the vertebrate host (different serotypes), the orbiviruses have maintained a significant level of conservation in OC1 that is related to vector group. Despite much higher identity levels in their conserved genes and proteins, WALV and MUDV share only 67.89%/69.22% aa/nt identity in Seg-2/VP2(OC1), confirming that they represent different serotypes (WALV-1 and WALV-2 respectively) within the same *Orbivirus* species.

## Discussion

The majority of the existing *Orbivirus* species were initially recognised as distinct ‘serogroups’, sharing common antigens detectable by complement-fixation (CF), agar gel immuno-diffusion (AGID), fluorescent antibody (FA) tests, or enzyme-linked immune-sorbent assays (ELISA) [50], [51]. However, these serological assays are relatively insensitive, can lack specificity and can show significant levels of cross-reactivity between closely related *Orbivirus* species [52]–[54]. Identification of new orbivirus isolates (by species and serotype) was also dependent on the availability of reference antisera for the virus species that have already been recognised.

The advent of more rapid and reliable sequencing methods for dsRNA virus genomes has generated data for reference strains of the individual virus species, that can be easily disseminated for phylogenetic comparisons [5], [19], [34], [37]. These data sets now represent a primary tool for the identification and classification of novel orbivirus isolates [25]–[27], [31], [55].

The orbivirus sub-core-shell ‘T2’, polymerase ‘Pol’ and outer-core ‘T13’ proteins are all highly conserved, with >83%, >73% and >73% aa identity respectively within a single *Orbivirus* species. Consequently, these proteins and their comparisons have been used as ‘markers’ for identification and classification of existing and novel orbivirus isolates [5], [18], [26], [31], [32]. The highest inter-species identity levels that have been detected in these conserved proteins are between the closely related and intensively studied species: *Bluetongue virus* and *Epizootic haemorrhagic disease virus* (with maximum aa identities of 80%, 73%, and 66% in T2, Pol and T13 protein respectively). The genes encoding these proteins have also served as targets for development of orbivirus genus-specific and species-specific RT-PCR assays that can be used for diagnosis and virus discovery [18], [29], [56]–[58].

Nucleotide variation of up to 31.6% (equivalent to >68.4% nt identity) has been observed in the serotype controlling VP2(OC1) gene between different BTV isolates within a single serotype, with a maximum of 71.5% nt identity between different types providing useful ‘bench marks’ for typing of isolates in other orbivirus species [32], [34]

Phylogenetic analysis of the three most conserved VP1, T2 and T13 proteins/genes and the highly variable OC1 protein of the orbiviruses, that are presented here show three distinct ‘clusters’ that correspond to the arthropod vectors used by each virus (Figure 2, 3 and 4), indicating that the orbiviruses have evolved through ‘*co-speciation*’ with their arthropod vectors [18], [33]. The TBOs and MBOs form distinct phylogenetic clusters originating from a common branch but are more closely related to each other than to the CBOs (Figure 4 and 5). The TBOs appear to provide an ancestral ‘root’ for the insect transmitted orbiviruses. The identification of a smaller OC1 proteins in all of the MBOs (when compared to the CBOs) and a much smaller protein (∼50%) in the TBOs suggests that significant evolutionary changes in OC1 may have played a significant role in the initial vector preference and evolutionary separation of these groups of viruses. The changes in size of the OC1 protein of the IBOs compared to the TBOs could be related to an historic concatemerization event [59] doubling the size of the protein during the evolution of the MBOs and CBOs.

Phylogenetic trees for the different genome segments/proteins of WALV, MUDV and WARV confirm that members of the same *Orbivirus* species cluster more closely together, while members of distinct species branch separately. The trees constructed for orbivirus Pol, T2 and T13 genes are almost entirely congruent, also showing similarities to the OC1 tree, although with higher levels of identity within each virus species. This suggests that functional interactions between the different viral proteins/RNAs may restrict the exchange of genome segments (reassortment) between virus species.

WALV and MUDV are indistinguishable in complement fixation tests and can reassort with high frequency [60], demonstrating that they belong to the same *Orbivirus* species - *Wallal virus*. The data presented here, showing that WALV and MUDV share >99%/90% aa/nt identities in their conserved Pol, T2 and T13 proteins support this conclusion (Table 3). However, WALV and MUDV can be distinguished in serum neutralization tests [60], [61] and are therefore regarded as distinct serotypes - WALV-1 and WALV-2 respectively. This is supported by the data presented here, showing that they share <68%/70% aa/nt identity in outer capsid protein VP2(OC1).

The utility of sequence data for phylogenetic analysis of viral pathogens, their classification, as well as diagnostic test and vaccine development, suggests that full-genome sequencing is becoming an accepted standard for molecular epidemiological studies. It will therefore be useful to generate a full-genome sequence database for all orbivirus isolates, to help elucidate relationships, determine the frequency and significance of gene exchange (reassortment), the mechanisms involved in virus movement and emergence, and their impact on disease in vertebrate populations. The generation of full-genome sequences for reference strains of *Wallal virus* and *Warrego virus*, completes a genome data set for reference strains of the *Culicoides* borne *Orbivirus* species, including BTV, AHSV, EHDV, EUBV, PALV and EEV. The sequences generated in this study will not only help to identify novel Wallal and Warrego viruses, but will also provide a useful tool for identification and study of other orbiviruses, particularly other CBOs and those affecting marsupials.

Full genome references sequences are now available for fourteen of the twenty two *Orbivirus* species recognised by ICTV. These data will provide a basis for development of relevant diagnostic assays, and support molecular epidemiology/evolutionary studies, to promote our understanding of orbivirus disease in marsupials and other host groups.

## Supporting Information

File S1 **Table S1 and Figure S1.** Table S1. Nucleotide accession numbers for sequences used in phylogenetic analysis. Figure S1. Unrooted Neighbour-joining phylogenetic tree comparing orbivirus T2 proteins.
(DOCX)
