# Supplementary material for: Full Genome Characterization of the Culicoides-Borne Marsupial Orbiviruses: Wallal Virus, Mudjinbarry Virus and Warrego Viruses
Source: PLoS One. 2014 Oct 9;9(10):e108379. doi: 10.1371/journal.pone.0108379 (PMC4191977; doi:10.1371/journal.pone.0108379)
Supplement: File S1 — Table S1 and Figure S1. Table S1. Nucleotide accession numbers for sequences used in phylogenetic analysis. Figure S1. Unrooted Neighbour-joining phylogenetic tree comparing orbivirus T2 proteins. (DOCX) [file pone.0108379.s001.docx]

**Supplementary data**

**Table S1: List of nucleotide accession numbers used for sequence and phylogenetic analysis**

| **Species** | **Virus (Isolate)** | **VP1** | **OC1** | **T2** | **CaP** | **NS1** | **OC2** | **T13** | **NS2** | **VP6** | **NS3** |
| --- | --- | --- | --- | --- | --- | --- | --- | --- | --- | --- | --- |
| ***Bluetongue virus*** | **BTV-1e (IND1992/01)** | JQ282770 | AJ585111 | JQ282771 | JQ282772 | JQ282773 | AJ586659 | JQ282774 | JQ282775 | JQ282776 | JQ282777 |
|  | **BTV-8w (NET2006/04)** | AM498051 | AM498052 | AM498053 | AM498054 | AM498055 | AM498056 | AM498057 | AM498058 | AM498059 | AM498060 |
|  | **BTV-25** | GQ982522 | EU839840 | GQ982523 | GQ982524 | EU839841 | EU839842 | EU839843 | EU839844 | EU839845 | EU839846 |
|  | **BTV-26**  **(KUW2010/02)** | JN255156 | HM590642 | HM590643 | JN255157 | JN255158 | JN255159 | HM590644 | JN255160 | JN255161 | JN255162 |
| ***African horse sickeness virus*** | **AHSV-1**  **(HS29/62)** | FJ183364 | FJ183365 | FJ183366 | FJ183367 | FJ183368 | FJ183369 | FJ183370 | FJ183371 | FJ183372 | FJ183373 |
| ***Epizootic haemorrhagic disease virus*** | **EHDV-1w**  **(USA1955/01)** | AM744977 | AM744978 | AM744979 | AM744980 | AM744981 | AM744982 | AM744983 | AM744984 | AM744985 | AM744986 |
|  | **EHDV-2e (JAP1959/01)** | AM745077 | AM745078 | AM745079 | AM745080 | AM745081 | AM745082 | AM745083 | AM745084 | AM745085 | AM745086 |
| ***Equine encephalosis virus*** | **EEV**  **(HS103/06)** | FJ183384 | FJ183385 | FJ183386 | FJ183387 | FJ183388 | FJ183389 | FJ183391 | FJ183390 | FJ183392 | FJ183393 |
| ***Eubenangee viurs*** | **EUBV**  **(AUS1963/01)** | JQ070376 | JQ070377 | JQ070378  AF530087^*^ | JQ070379 | JQ070380 | JQ070381 | JQ070382 | JQ070383 | JQ070384 | JQ070385 |
|  | **TILV**  **(AUS1978/03)** | JQ070366 | JQ070367 | JQ070368 | JQ070369 | JQ070370 | JQ070371 | JQ070372 | JQ070373 | JQ070374 | JQ070375 |
| ***Palyam virus*** | **CHUV** | AB018086 | AB014725 | AB014728 | AB018087 | AB018089 | AB014726 | AB014727 | AB018090 | AB018088 | AB018091 |
|  | **DAGV**  **(B8112)** | --- |  | AF530085 | --- | --- | --- | --- | --- | --- | --- |
| ***Umatilla virus*** | **UMAV**  **(USA1969/01)** | HQ842619 | HQ842621 | HQ842620 | HQ842623 | HQ842622 | HQ842624 | HQ842626 | HQ842625 | HQ842627 | HQ842628 |
|  | **SLOV** | NC_012754 | --- | NC_012755 | --- | --- | --- | --- | --- | --- | --- |
| ***Peruvian horse sickness virus*** | **PHSV** | DQ248057 | DQ248059 | DQ248058 | DQ248060 | DQ248064 | DQ248061 | DQ248063 | DQ248065 | DQ248062 | DQ248066 |
| ***Corriparta virus*** | **CORV** | KC853042 | KC853044 | KC853043,  AF530086^*^ | KC853045 | KC853046 | KC853047 | KC853049 | KC853048 | KC853050 | KC853051 |
|  | **CMPV** | --- | --- | EU789391^*^ | EU789392^*^ | --- | EU789393^*^ | EU789394^*^ | --- | EU789395^*^ | --- |
| ***Yunnan orbivirus*** | **YUOV** | AY701509 | AY701511 | AY701510 | AY701512 | AY701513 | AY701514 | AY701516 | AY701515 | AY701517 | AY701518 |
|  | **MPOV** | --- | EF591621 | EF591620 | --- | --- | --- | --- | --- | --- | --- |
| ***Great Island virus*** | **GIV**  **(CanAr 42)** | HM543465 | HM543469 | HM543466 | HM543467 | HM543468 | HM543470 | HM543471 | HM543472 | HM543473 | HM543474 |
|  | **BRDV** | --- | --- | M87875 | --- | X82599 | M58030 | M87876 | --- | --- | M83197 |
|  | **LIPVh**  **(CzArLip 91)** | HM543475 | --- | HM543476 | --- | --- | HM543477 | --- | --- | --- | --- |
|  | **TRBVh** | HM543478 | --- | HM543479 | --- | --- | HM543480 | --- | --- | --- | --- |
|  | **KEMVh**  **(EgAn 1169-61)** | HM543481 | --- | HM543482 | --- | --- | HM543483 | --- | --- | --- | --- |
|  | **TRBV** | HQ266581 | HQ266585 | HQ266582 | HQ266583 | HQ266584 | HQ266586 | HQ266588 | HQ266587 | HQ266589 | HQ266590 |
|  | **KEMV** | HQ266591 | HQ266594 | HQ266592 | HQ266593 | HQ266595 | HQ266596 | HQ266598 | HQ266597 | HQ266599 | HQ266600 |
| ***St Croix river virus*** | **SCRV** | NC_005997 | NC_005998 | NC_005999 | NC_006000 | NC_006002 | NC_006001 | NC_006004 | NC_006003 | NC_006005 | NC_006006 |
| ***Warrego virus*** | **WARV**  **(Ch 9935)** | --- | --- | AF530083^*^  EF213555^*^ | --- | --- | --- | --- | --- | --- | --- |
| ***Wallal virus*** | **WALV**  **(Ch 12048)** | --- | --- | AF530084^*^ | --- | --- | --- | --- | --- | --- | --- |
| ***Wongorr virus*** | **WGRV**  **(V5080, V195, V199, mrm13443)** | --- | --- | U56989^*^,  U56990^*^,  U56991^*^,  U56992^*^ | --- | --- | --- | --- | --- | --- | --- |
|  | **PARV** | --- | --- | U56993^*^ | --- | --- | --- | --- | --- | --- | --- |
|  | **PIAV** | --- | --- | U56994^*^ | --- | --- | --- | --- | --- | --- | --- |
| **Pata virus** | **PATAV**  **(CAF1968/01)** | JQ070386 | JQ070387 | JQ070388 | JQ070389 | JQ070390 | JQ070391 | JQ070393 | JQ070392 | JQ070394 | JQ070395 |
| **California mosquito pool virus** | **CMPV** | --- | --- | EU789391^*^ | EU789392^*^ | --- | EU789393^*^ | EU789394^*^ | --- | EU789395^*^ | --- |
| **Sathuvachari virus** | **SVIV**  **(IAn66411)** | KC432629 | KC432630 | KC432631 | KC432632 | KC432636 | KC432633 | KC432635 | KC432637 | KC432634 | KC432638 |

^*^only partial sequences are available. Pata virus (represented in grey colour) represents novel species in the genus *Orbivirus* (Belaganahalli et al., 2012). **Pol**=Polymerase, **OC1**=Outer capsid protein 1 (VP2 of BTV), **T2**=Inner core protein (T2 symmetry), **Cap**= Capping enzyme, **Tup**=Tubule forming protein or Tubular protein (NS1), **OC2**=Outer capsid protein 2 (VP5 of BTV), **T13**=Outer core protein (T13 symmetry), **ViP**=Viral inclusion body protein (NS2), **Hel**=Helicase protein.

**BTV26**

**BTV25**

68

**BTV8w**

**BTV1e**

100

100

**PATAV**

**EHDV2e**

**EHDV1w**

100

90

100

**MUDV●**

**WALV●**

**WALV**

*****

100

**EUBV**

*****

**TILV**

**EUBV**

100

100

98

**WARV**

*****

**WARV 69●**

**WARV5080**

*****

100

95

**DAGV**

*****

**CHUV**

**AHSV1**

95

**EEV**

100

**WGRV**

*****

**PICV***

**PARV***

**WGRV195***

**WGRV199***

100

**SLOV**

**UMAV**

100

67

**CMPV**

**CORV**

*****

**CORV**

100

**PHSV**

**YUOV**

**MPOV**

100

**SVIV**

**GIV**

**BRDV**

100

**KEMV**

**LIPV**

**TRBV**

100

100

100

**SCRV**

0.1

**T2=VP3**

**T2=VP2**

***Culicoides***

**Mosquitoes**

**Ticks**

**Figure S1: Unrooted neighbour-joining phylogenetic tree comparing orbivirus T2 protein sequences.**

The tree was constructed using distance matrices, generated using the p-distance determination algorithm in MEGA 5 (1000 bootstrap replicates) (Tamura et al., 2011). The numbers at nodes indicate bootstrap confidence values after 1000 replications. The WALV, MUDV and WARV isolates characterised in this study are indicated by a green button symbol. Full names of virus isolates and accession numbers of T2 protein sequences used for comparative analysis are listed in Table S1 (supplementary data). ‘e’ and ‘w’ after serotype number indicate eastern and western strains, respectively.

(Tamura, K., Peterson, D., Peterson, N., Stecher, G., Nei, M., Kumar, S., 2011. MEGA5: Molecular Evolutionary Genetics Analysis using Maximum Likelihood, Evolutionary Distance, and Maximum Parsimony Methods. Mol Biol Evol 28, 2731-2739)
